# Evolutionary obstetrics

**DOI:** 10.1093/emph/eou025

**Published:** 2014-11-11

**Authors:** Karen R. Rosenberg, Wenda R. Trevathan

**Affiliations:** ^1^Department of Anthropology, University of Delaware, Newark, DE 19716, and ^2^Department of Anthropology, New Mexico State University, Las Cruces, NM 88003

## Definition and background

Birth is a challenging and perhaps painful process for primates that have large heads relative to their body size. This includes our own species, but for humans, the pelvic changes resulting from the origin of bipedalism 5–7 million years ago restructured the pelvis, altered the birth mechanism and further limited the size of the birth canal. Additional challenges to the birth process occurred with dramatically increased brain size starting about 2 million years ago [1].

The bipedal birth canal is ‘twisted’ in the middle with the inlet and outlet perpendicular. In between these two planes is the midplane, which usually presents the smallest diameter. The changing shape typically involves rotations of the infant in order to pass through the three pelvic planes to emerge with the occiput anterior (OA), facing away from the mother. These challenges place a selective advantage on having assistance for guiding the baby out, clearing the face and nose and dealing with a wrapped umbilical cord. Worldwide it is rare for women to give birth alone. Western medical perspectives on childbirth view it as sufficiently risky that it must take place in a clinical setting.


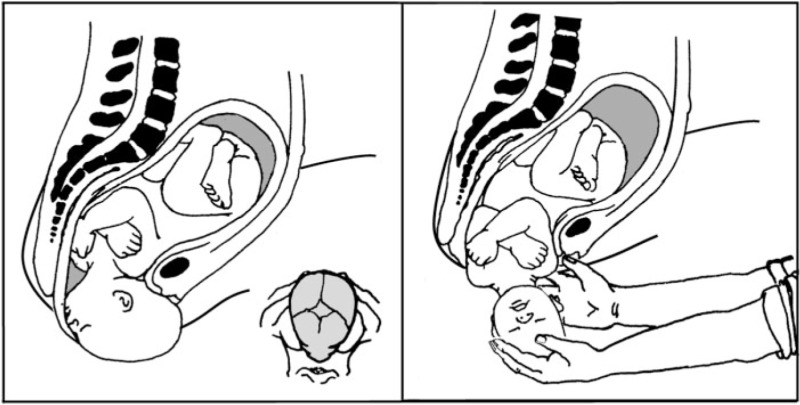


## Examples in human biology and public health

Two evolutionary legacies that impact childbirth today are the potential for obstructed labor and the selective premium on having assistance at birth. Obstructed labor accounts for 12% [2] of maternal mortality worldwide and includes both cephalopelvic disproportion and shoulder dystocia. The dimensions of the neonatal head and maternal pelvis are so close that passage through the birth canal is often constrained. In addition, our broad shoulders, a legacy of our ape ancestry, can result in dystocia. Although the incidence of obstructed labor has certainly increased since the origins of agriculture [3] and with modern obstetric interventions such as the lithotomy position, large heads and broad shoulders probably posed challenges to birth in our earliest human ancestors and account for a significant portion of necessary surgical deliveries today.

Most nonhuman primates give birth with the occiput posterior, allowing the mother to easily guide the infant up toward her chest. With the anatomical changes that accompanied bipedalism, humans typically emerge OA. Simply having another person present to guide the infant out reduces the risks associated with OA deliveries [1].

## Examples in clinical medicine

For most of history and in many parts of the world today, the most common delivery position is upright in a squatting or standing position. This widens the birth canal and enables gravity to work with contractions to expel the baby. Replacing the lithotomy position with supported squatting incorporates a beneficial practice from the past into contemporary birth.

A desire for social and emotional support during birth reflects the evolutionary context in which humans have given birth for at least the history of our species [1]. In many modern medical contexts there is a mismatch between the evolved emotional needs of women and the clinical environments in which many births take place; this can be corrected with social and emotional support during labor and delivery such as that offered by doulas.
